# In the Shadow of Hemagglutinin: A Growing Interest in Influenza Viral Neuraminidase and Its Role as a Vaccine Antigen

**DOI:** 10.3390/v6062465

**Published:** 2014-06-23

**Authors:** Teddy John Wohlbold, Florian Krammer

**Affiliations:** 1 Department of Microbiology, Icahn School of Medicine at Mount Sinai, One Gustave L. Levy Place, Box 1124, New York, NY 10029, USA; E-Mail: teddyjohn.wohlbold@mssm.edu; 2 Graduate School of Biological Sciences, Icahn School of Medicine at Mount Sinai, New York, NY 10029, USA

**Keywords:** influenza virus, neuraminidase, influenza virus vaccine, universal influenza virus vaccine, sialidase, cross-protection

## Abstract

Despite the availability of vaccine prophylaxis and antiviral therapeutics, the influenza virus continues to have a significant, annual impact on the morbidity and mortality of human beings, highlighting the continued need for research in the field. Current vaccine strategies predominantly focus on raising a humoral response against hemagglutinin (HA)—the more abundant, immunodominant glycoprotein on the surface of the influenza virus. In fact, anti-HA antibodies are often neutralizing, and are used routinely to assess vaccine immunogenicity. Neuraminidase (NA), the other major glycoprotein on the surface of the influenza virus, has historically served as the target for antiviral drug therapy and is much less studied in the context of humoral immunity. Yet, the quest to discern the exact importance of NA-based protection is decades old. Also, while antibodies against the NA glycoprotein fail to prevent infection of the influenza virus, anti-NA immunity has been shown to lessen the severity of disease, decrease viral lung titers in animal models, and reduce viral shedding. Growing evidence is intimating the possible gains of including the NA antigen in vaccine design, such as expanded strain coverage and increased overall immunogenicity of the vaccine. After giving a tour of general influenza virology, this review aims to discuss the influenza A virus neuraminidase while focusing on both the historical and present literature on the use of NA as a possible vaccine antigen.

## 1. Influenza Virus: Epidemiology and Basic Virology

The influenza virus has historically plagued the human race, causing yearly seasonal epidemics and—less frequently—global pandemics, as evidenced by the recent outbreak of H1N1 (originally termed “swine flu”) in 2009. The virus has likely co-existed and co-evolved with human beings for millennia; in fact, medical historians suspect that it was one of the main etiological culprits of the “Cough of Perinthus”, an epidemic of upper respiratory tract infection described in detail by Hippocrates in 412 BC [1]. The 20th century alone witnessed the occurrence of three influenza virus pandemics, the deadliest of which was the Spanish Flu of 1918, which claimed the lives of 50–100 million people, or 3%–5% of the world population at the time [2]. From an alternative perspective, the 1918 H1N1 virus killed an equivalent amount of people in one year (from September 1918 to March 1919) as did HIV over the span of its first 25 years of circulation in the human population [3]. The current mean number of influenza-related deaths is difficult to calculate due to the extreme variability in length and severity of the flu season; however, the World Health Organization (WHO) estimates that seasonal influenza epidemics annually cause 250,000–500,000 deaths worldwide [4]. What is more, influenza epidemics are associated with tremendous disease burden, costing the United States $87.1 billion in the financial year of 2003 alone, when factoring in the cost of hospitalization, missed work, and other parameters [5].

Influenza viruses characteristically house a single-stranded, negative-sense, segmented RNA genome, which exists in the infectious particle as ribonucleoprotein (RNP) complexes and is further contained within a host-cell derived lipid bilayer. Categorized into the *Orthomyxoviridae* family, influenza viruses can be further subdivided into three genera—*Influenzavirus A*, *B*, and *C* [6]. While viruses from all three genera have been shown to infect humans, only influenza A and B viruses substantially contribute to seasonal epidemics [7]. Furthermore, while influenza B viruses may play a significant role in pediatric influenza cases, surveillance data from the Centers for Disease Control (CDC) has revealed that they tend to cause only a minority (<20%) of total influenza cases per year [8].

**Figure 1:**
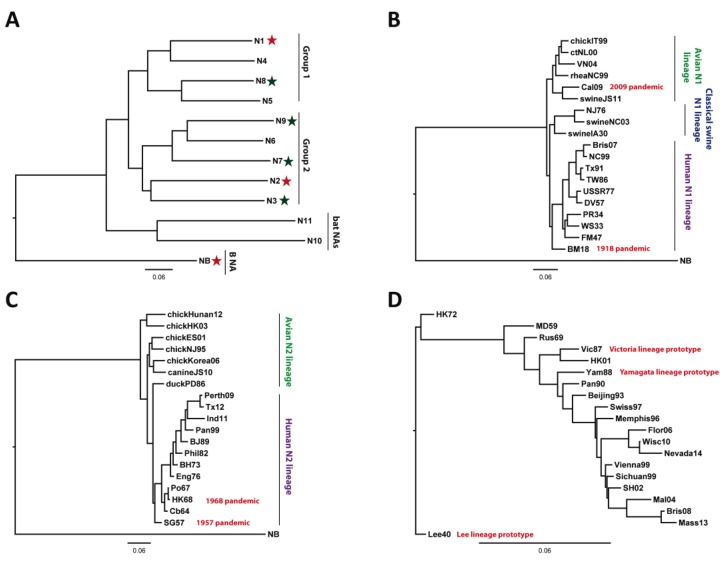
Phylogenetic relationships of influenza virus neuraminidase proteins. (**A**) Phylogenetic tree of influenza A and B NAs including the recently isolated N10 and N11 subtypes for which no NA activity has been reported. NA subtypes that circulate in humans are indicated by red stars. Subtypes that occasionally cause human infections are indicated by green stars. (**B**) Phylogenetic tree of N1 NAs. N1 NAs form three lineages, the avian lineage, which includes the NA of the 2009 pandemic H1N1 virus, the classical swine lineage and the now extinct human lineage. (**C**) Phylogenetic tree of N2 NAs. N2 forms an avian and a human phylogenetic lineage. The latter one split of from the avian lineage with the 1957 H2N2 pandemic strain and continued to circulate as H3N2 in 1968 when H2N2 viruses disappeared. (**D**) Phylogenetic tree of influenza B virus NAs. NAs of prototypic Lee, Yamagata and Victoria strains are indicated. It is of note that the B NAs do not split into the Victoria87 and Yamagata88 lineages like B HA sequences. However, there seems to be a recent split into three distinct lineages with one (HK01-like, 2001 isolate) clustering closest with the NA of the Victoria-lineage prototype from 1987. Scale bars represent a 6% difference in amino acid identity. Trees in **A**–**C** were rooted with B NA, the tree in D was rooted using the B Lee ancestral sequence. All trees were built using the “default” setup of ClustalW and were visualized using FigTree. GenBank/GISAID accession numbers for all sequences used can be found in the supplementary material file (Table S1).

Unlike influenza B and C—which are thought to only replicate in human hosts—influenza A has been shown to infect and replicate in a much broader variety of non-human species (including poultry, sea mammals, pigs, horses and—more recently—New World bats) [3]. This wide host range has allowed influenza A viruses to acquire much more genetic diversity over evolutionary time compared to counterpart viruses from other *Orthomyxoviridae* genera. Modern taxonomy systems classify existing and emergent influenza A virus subtypes based on the sequence and antigenicity divergence of the virus’ two major surface glycoproteins, hemagglutinin (HA) and neuraminidase (NA), which display the most amino acid sequence diversity out of all influenza virus proteins [3,6]. Since 2009, two additional HA and NA subtypes have been discovered in New World bat species, meaning a total of 18 HA subtypes (H1-18) and 11 NA subtypes (N1-11) have been found in nature thus far (Figure 1) [9]. Only a subset of HAs (H1, H2, and H3) and NAs (N1 and N2) are known to naturally circulate in the human population, although H5, H6, H7, H9, H10, N3, N7, N8, and N9 have been found in human cases mostly associated with poultry outbreaks (Figure 1) [10,11,12,13,14,15,16,17,18]. As viruses of all known subtypes (except the two most recently discovered, H17N10 and H18N11) are maintained in aquatic birds, it is thought that these species are the natural evolutionary reservoir of the influenza A virus [3].

## 2. Current Therapeutic/Prophylactic Approaches

Annual vaccine administration (either with the intramuscular trivalent inactivated vaccine [TIV] or the intranasal live attenuated vaccine [LAIV]) continues to be the mainstay of preventative treatment for influenza virus infection in humans [19]. Antiviral drugs, such as M2 ion channel inhibitors (amantadine and rimantadine) and NA inhibitors (oseltamivir and zanamivir) have been indicated to treat influenza virus infection but continue to have serious limitations. For instance, they must be used within 24–48 h after the onset of infection and cannot be used in high-risk patients (those less than 1 year of age or with renal or liver failure) [13]. Furthermore, influenza virus strains exhibit marked resistance to antivirals; in fact, circulating influenza A virus strains have developed such wide resistance to M2 ion channel inhibitors that these drugs are no longer officially recommended for the treatment of influenza in the United States by the Advisory Committee on Immunization Practices (ACIP) [20].

Despite the availability of the influenza virus vaccine, seasonal epidemics—and the ever-looming threat of an emergent pandemic—continue to pose a challenge to human health. This is rooted in the ever-changing antigenic diversity of the influenza A virus, which allows it to escape neutralizing antibodies, and ultimately results in vaccine antigenic mismatch—when the strains included in the annually administered vaccine (based on WHO surveillance data) do not match those that are currently circulating [19]. Furthermore, current influenza virus vaccines take months to manufacture and are thus often rendered obsolete by emerging strains. Understandably, the need for novel vaccine strategies in the treatment of influenza is clear [19].

The quest for developing a more effective—and perhaps universal—influenza vaccine has led to a variety of novel approaches, including but not limited to the use of purified, recombinant HA expressed by baculovirus-infected insect cells [21], adjuvanted inactivated vaccines [22], recombinant virus-like particles (VLPs) [23,24], and vaccine candidates designed to elicit broadly-reactive, stalk-based HA antibodies [25]. Indeed, Flublok^®^—a vaccine composed of recombinant HA proteins from three different influenza strains and expressed in Sf9 insect cells—was recently approved for use by the FDA in January 2013.

Most of the current approaches to vaccine design focus on measuring the antibody response to influenza A HA, the predominant homotrimeric glycoprotein located on the surface of the virus. This is expected given the two known roles of HA in the lifecycle of influenza virus infection: HA mediates attachment to host cell receptors (by binding to α2–6 and α2–3 linked sialic acid residues) and mediates fusion of the viral endosome with the lysoendosomal membrane following a pH-dependent conformational change [6]. Presumably, anti-HA antibodies are often neutralizing, and can even be sterilizing. The humoral response against HA has been thoroughly studied, and the antigenic surfaces on the glycoprotein have been mapped to 4–5 sites that are exclusively located in the globular head domain [3,26]. Amino acid substitutions in these regions may lead to antigenic drift, and recently it was shown that escape mutations may be correlated with increased receptor binding avidity [27].

## 3. Influenza Neuraminidase: Structure, Function, and Role in Influenza Virus Infection

In the scope of influenza virus research, relatively little attention is given to the neuraminidase, and while the use of neuraminidase as a vaccine antigen is studied by only a small pocket of investigators, recent evidence has reignited interest in the potential use of NA in vaccine design as well as the ability of NA-directed antibodies to confer protection against a range of influenza virus subtypes [28]. Indeed, the possibility of NA-based protection has been recognized by the international vaccine community, and the proposal to somehow standardize the amount of NA in vaccine formulations has been entertained at past WHO conferences on influenza virus vaccine development [29,30].

**Figure 2:**
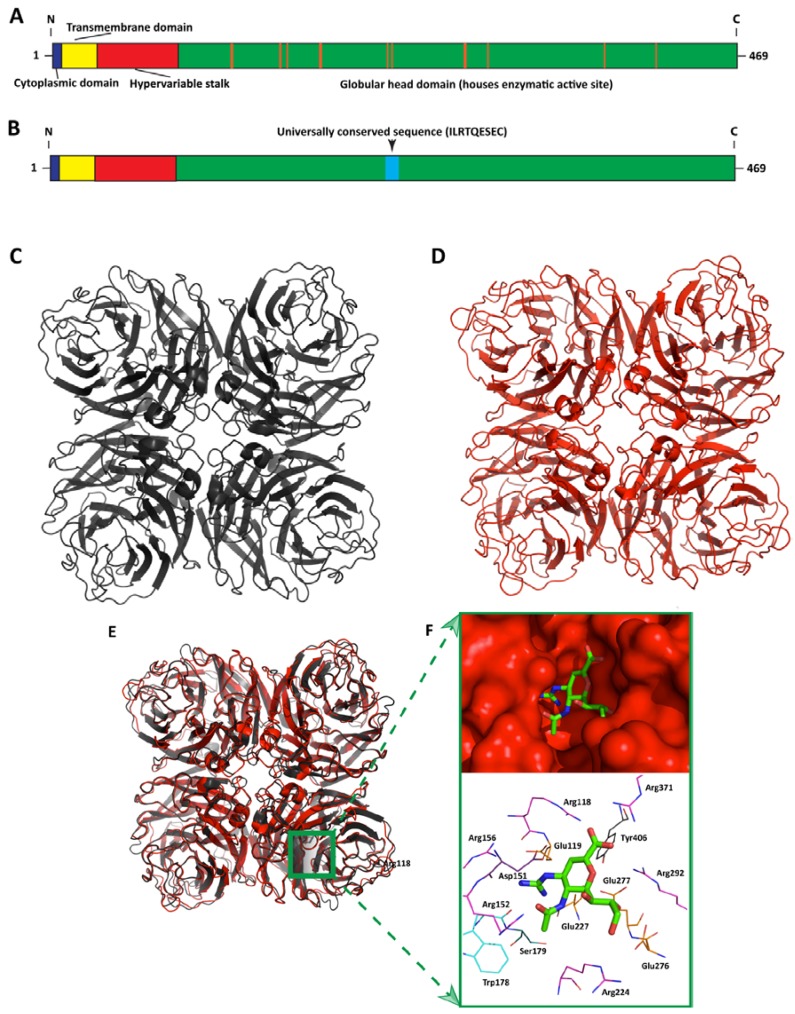
The 3D structure of Influenza A Virus NA is highly conserved. (**A**) Schematic of the NA protein (to scale) showing the cytoplasmic, transmembrane, hypervariable stalk, and globular head domains, using amino acid residue numbering from the NA of A/Brevig Mission/1/1918 (H1N1). Orange lines represent the positions of active site residues that form contacts with zanamivir (as determined from the crystal structure of the active site shown in panel 2F). (**B**) Highlighted in light blue is the position of the 9-residue sequence ILRTQESEC, which is universally conserved among all known NA subtypes (again, N1 numbering from A/Brevig Mission/1/1918) [39]. NA displays a remarkably conserved 6-bladed propeller structure. Each blade is made up of four anti-parallel beta sheets stabilized by disulfide bonds and connected by loops of varying length. (**C**) 3D crystal structure of the globular head domain of the NA from A/Brevig Mission/1/1918 (H1N1) (PDB ID: 3B7E), shown as a tetramer. (**D**) 3D crystal structure of the globular head domain of the NA from A/Tanzania/205/2010 (H3N2) (PDB ID: 4GZQ) [40], shown as a tetramer. (**E**) Despite the amino acid sequence differences across NA subtypes, the 3D structure tends to be conserved, as evidenced by the aligned structural overlay of the N1 and N2 from previous figure panels, 2C and 2D. There is one enzymatic active site per NA monomer, although NA is thought to be only enzymatically active as a tetramer. (**F**) Zoomed-in view of one of the four identical active sites of the NA tetramer (A/Brevig Mission/1/1918) complexed with zanamivir (the approximate corresponding location on the 3D structure in panel 2E is indicated by a green box). The electron density of NA is shown as a solid red contour map (**top** panel). Residues that make chemical contacts with zanamivir in the crystal structure are shown as color-coded, labeled lines (**bottom** panel).

The neuraminidase has not always stood in the shadow of the hemagglutinin in the history of influenza virus research. In fact, shortly after George Hirst discovered the presence of what was then termed the “receptor destroying enzyme” in the 1940s, scientists were quick to realize that the neuraminidase could be targeted as a therapeutic approach to fight influenza virus infection [31]. In 1948, MacFarlane Burnet wrote, “An effective *competitive poison* for the virus enzyme might be administered which, when deposited on the mucous film lining the respiratory tract would render this an effective barrier against infection...” [32]. After it was discovered that the globular head domain of neuraminidase could be stably isolated from the full-length polypeptide by pronase treatment [33], it took approximately 20 years for the protein to be successfully purified and crystalized. The structure of an influenza A virus N2, which was shown to be a homotetramer, was subsequently published in *Nature* in 1983 [34], two years after that of a hemagglutinin [35]. Even before the structure of the NA was published, transition state inhibitors of the enzyme had been identified and shown to be effective in preventing viral replication in tissue culture; however such compounds failed to prevent disease in animals [36]. The crystal structure allowed for more targeted drug design, however, and in 1993, the first effective inhibitor of NA, a 2-deoxy-2,3-didehydro-*N*-acetylneuraminic acid (DANA) derivative (Figure 2F), was discovered and eventually marketed under the trade name Relenza (generic name: zanamivir) in 1999 [37,38].

Not unlike those of the HAs, NA sequences phylogenetically can be classified into two groups with Group 1 encompassing N1, N4, N5, and N8 and Group 2 encompassing N2, N3, N6, N7, and N9 (Figure 1A) [38]. The novel NA subtypes recently isolated in bats (N10 and N11) seem to be much more distantly related to existing subtypes and display no apparent sialidase activity, possibly warranting the formation of a third group (Figure 1A) [9]. As shown on the phylogram in Figure 1B, N1 has diverged into three species-defined lineages over time—avian, swine, and the now-extinct human lineage (which was replaced in humans by the emergence of the pandemic H1N1 strain in 2009, but which continues to circulate in swine). Pandemic N1 falls phylogenetically into the avian N1 lineage. N2 has diverged into two lineages—avian and human, both of which are currently circulating (Figure 1C). Understandably, the neuraminidase from influenza B (which is thought to almost exclusively infect humans) does not diverge into similar species-defined lineages (Figure 1D) and interestingly, its evolution seems to be independent from that of influenza B HA, which clusters into the Victoria and Yamagata lineages.

The NA glycoprotein is a type II integral membrane protein composed of four identical polypeptides arranged in a non-covalently bound homotetramer; each monomer contains approximately 470 amino acid residues and is organized into four domains—an *N*-terminal cytoplasmic domain, a hydrophobic transmembrane region, a thin hypervariable stalk, and a globular head domain that houses the enzymatic active site (Figure 2A) [38]. Although the enzymatic active site of each individual NA monomer appears to be independently located in the crystal structure, isolated monomeric forms of the protein lack enzymatic activity [41,42,43]. Furthermore, unlike the stalk region, which can readily accommodate mutations, the enzymatic active site is extremely conserved among the majority of subtypes [44]. In general, NA amino acid sequences can display tremendous sequence diversity across subtypes (Figure 1A). One review paper compared amino acid sequences between subtypes and found as much as a 66.8% identity (between an N5 and N8 subtype) and as little as 37.3% identity (between an N5 and N9 subtype) [44]. By comparison, as much as a 20% amino acid sequence divergence was seen within subtypes for both the N1 and N2 subtypes (Figure 1B,C) [44]. Nevertheless, the structure of the NA globular head domain is remarkably conserved across subtypes (Figure 2C–E).

The potential for the conserved enzymatic site to serve as a universal vaccine epitope was postulated even in the early studies of the glycoprotein, although it was realized that antibody binding to this epitope could be disrupted by mutations in amino acids surrounding the active site [45]. However, one group recently identified an eight amino acid long sequence in the NA active site that is 100% conserved in all influenza A virus subtypes as well as in the NAs of influenza B strains [46,47]. This amino acid sequence, “ILRTQESEC”, is located between residues 222 and 230 (N2 numbering) in the enzymatic active site (Figure 2B). Using the Enzyme-Linked Lectin Assay (ELLA), the group showed that a rabbit monoclonal antibody (mAb) raised against this linear epitope was able to inhibit the enzymatic activity of practically all influenza A NA subtypes N1-9 (See Table 2, reference [46]). Shortly after, the same group demonstrated that this mAb had the potential to broadly inhibit influenza B virus strains from both the Victoria and Yamagata lineages *in vitro*, as well as drug resistant influenza B virus mutants [47]. While the inhibitory potential of mAbs raised to this universally conserved enzymatic site seems promising, it is unknown—and probably unlikely—that these antibodies are induced by natural influenza virus infection or by current vaccination strategies. Furthermore, while the rabbit mAb heretofore described by Doyle *et al.* displays a wide spectrum of heterosubtypic NA enzymatic inhibition, its potency is an order of magnitude less, in general, than recently described mouse mAbs raised against the NA of seasonal H1N1 [48]. However, it is difficult to draw strong conclusions from this comparison, as the mouse mAbs do not necessarily target the same conserved epitope and have only been shown to inhibit the N1 subtype. These antibodies characterized by Wan *et al.* are nevertheless exciting and revisited in a later section.

**Figure 3:**
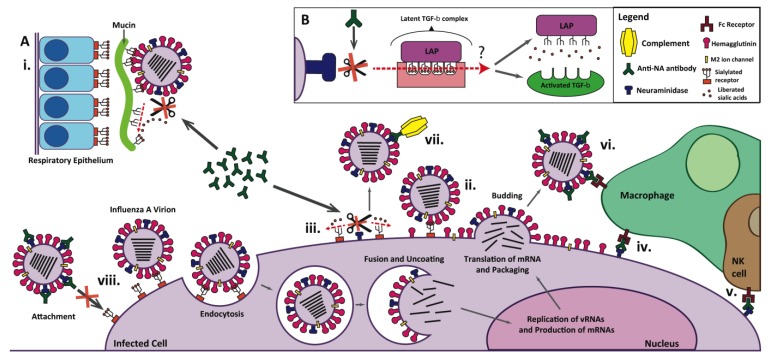
NA has various roles in influenza virus infection. (**A**) Upon entering the respiratory tract, influenza virions are often trapped in a protective layer of mucin (**i**) which contains sialylated decoy receptors (terminal sialic acids represented as little orange balls). NA enzymatically cleaves off these sialic acid residues and allows virus particles to penetrate the mucinous layer and access the underlying respiratory epithelium. The enzymatic activity of NA is represented by scissors and red dashed arrows. (**ii**) Nascent influenza virions remain attached to the host cell and to neighboring viruses (by the binding of HA to sialylated receptors) until freed by NA, which cleaves off sialic acid residues from host cell receptors (**iii**). While antibodies directed to NA are not neutralizing, they may bind to NA and inhibit each of its enzymatic functions (indicated by red X’s). Anti-NA antibodies bound to the surface of infected cells may aid in their recognition and clearance by immune effector cells such as macrophages (**iv**) and natural killer (NK) cells (**v**), in a process known as antibody-dependent cell-mediated cytotoxicity (ADCC). Anti-NA antibodies bound to viral particles may mediate the direct uptake of virions by macrophages (**vi**), or allow for the binding and activation of the complement system (**vii**), in a process known as complement-dependent cytotoxicity (CDC). Finally, it has been speculated that anti-NA antibodies bound to influenza virions may, by steric hindrance, interfere with the binding of HA to sialylated host cell receptors and, thus, prevent viral attachment and entry (**viii**) [59]. (**B**) Influenza A NA has been shown to enzymatically activate TGF-β, which normally exists in a latent form bound to the latency-associated peptide (LAP). While the exact mechanism of this activation is unknown, it is thought that NA cleaves off sialic acids from the LAP, causing it to dissociate from TGF-β. Theoretically, antibodies may also block this process.

**Figure 4:**
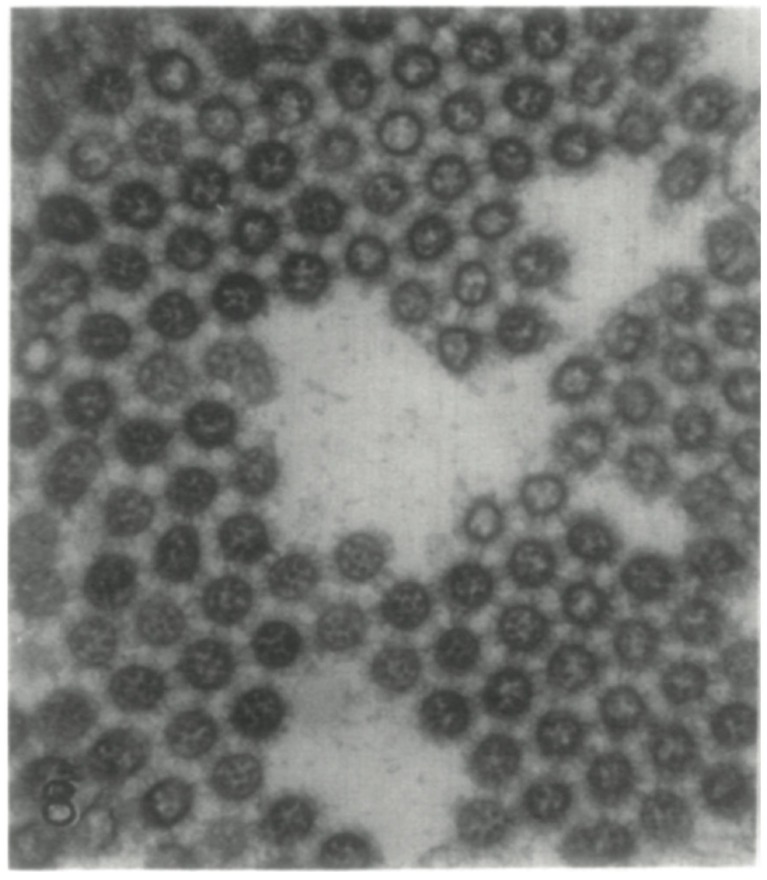
NA is needed for the successful detachment of nascent influenza virus particles from host cells. Shown here is one of the original electron micrographs from the work of Peter Palese *et al.* (taken with permission from P Palese, Tobita, *et al.*, 1974), showing the aggregation of influenza virus particles in the absence of neuraminidase activity [49].

Crucial to its function, NA enzymatically cleaves α2-6 and α2-3 linked sialic acids (Figure 3Aiii). In most cases, NA is expendable to viral entry and instead is thought to aid in the detachment of nascent viral particles by cleaving sialic acid residues from newly formed HA molecules on the surface of host cells (Figure 3Aii) [28]. In the absence of NA activity, influenza viruses can infect and fully carry out one replication cycle, but progeny viruses remain aggregated on the host cell surface [49] and thus fail to spread to uninfected cells (Figure 4). Interestingly, one group was, by chance, able to recently rescue viruses that use NA—as opposed to HA—as the receptor binding protein, and—perhaps unexpectedly—found that the NA of these viruses retain partial sialidase activity. This challenges the classic school of thought that NA is expendable for viral entry into host cells [50]. Similarly, it is conceivable NA could serve as the receptor binding protein of the novel bat influenza viruses since their HAs do not seem to have affinity for any of the tested glycosylated receptors [9].

The enzymatic activity of NA is also thought to facilitate influenza virus infection by penetrating respiratory tract mucins and removing decoy receptors from the glycocalyx of epithelial cells (Figure 3Ai). Mucous lining the mammalian respiratory tract contains sialic acid linkages similar to those found on the surface receptors of epithelial cells; these are thought to bind to and trap influenza virions, preventing them from gaining access to the underlying epithelium and aiding in their ultimate clearance from the airway [51]. This phenomenon has not been conclusively demonstrated *in vivo* but has been examined using frozen human tracheal and bronchial tissues which have been sliced in a way to preserve the overlaying mucous coating of epithelial cells [52]. It has been shown that both H1N1 and H3N2 influenza virus subtypes directly bind to the preserved mucous layer and that this binding is disrupted either with prior addition of *Arthrobacter ureafaciens* sialidase (a bacterial sialidase) or sodium periodate (a chemical treatment that truncates terminal sialic acid side chains) [52]. In the same paper, a preparation of purified human salivary mucins, which the authors claim resemble the mucin composition of actual human tracheal submucosal glands, was able to reduce the percentage of influenza A virus infected cells when added to a monolayer of MDCK cells. Moreover, this effect was dose dependent (that is, increasing molar concentrations of sialic acids in the mucin preparation resulted in decreasing proportions of infected cells) and augmented by the addition of oseltamivir [52]. Interestingly, significant protection against the same H1N1 and H3N2 strains was not seen with purified swine submaxillary mucins, even though one of the tested H1N1 strains (A/SD/1/2009) is known to be of swine origin. Using mucins conjugated to magnetic streptavidin beads, the authors also showed that influenza A virus strains can directly cleave sialic acids; predictably, when the viruses were first incubated with oseltamivir, these sialic acids were not cleaved and the virus instead remained bound to the mucins [52]. One can easily imagine how the interaction between the influenza A virus neuraminidase and mucins of the respiratory tract influences the ease with which the virus infects the host.

A final interesting consequence of NA enzymatic activity on host immunity is the activation of transforming growth factor β (TGF-β), shown schematically in Figure 3B. The TGF-β superfamily is comprised of a group of regulatory cytokines that have been found to play vast and varied roles in immune modulation [53]. The mature TGF-β molecule is located extracellularly and normally exists in a latent form (latent TGF-β or LTGF-β), bound noncovalently to the latency-associated peptide (LAP) [53]. Though TGF-β must be freed from the LAP for activation, the precise mechanism of such activation remains unknown. Heat, extremes in pH, reactive oxygen species, chaotropic agents, as well as host and microbial proteases have been shown to activate latent TGF-β (LTGF-β) experimentally [54,55,56]. The full scope of TGF-β’s effect on the immune system has yet to be elucidated, but the cytokine’s crucial function appears to be the maintenance of tolerance through regulation of lymphocyte proliferation, differentiation, and survival. Normally, TGF-β tends to prevent the development of immunopathology and inflammation without compromising host immunity [53]. In fact, many parasites intentionally utilize this feature to create an immunoprivileged state within the host, which allows for the persistence of infection [56]. It has been known for some time that influenza virus infection can activate LTGF-β *in vitro* and *in vivo* [57], but more recently it has been directly shown that the influenza virus neuraminidase (specifically an N4 subtype neuraminidase purified from an H4N4 virus) can enzymatically cleave off sialic acid motifs from the LAP, thus activating LTGF-β (Figure 3B) [58]. Further studies from this paper revealed that most strains of influenza virus are capable of activating LTGF-β *in vitro*, with H5N1 subtypes being the least able to do so [58].

As of now, the complete significance of TGF-β’s activation by the influenza virus neuraminidase can only be speculated. While viral titers do not appear to be affected by the ability of the influenza virus to activate TGF-β [58], perhaps the virus somehow utilizes TGF-β regulation to reduce viral clearance to allow for a more persistent infection and more prolonged viral spread.

While anti-NA antibodies are thought to be non-neutralizing, they may conceivably alter any of the other previously mentioned roles of NA in influenza virus infection. Although they are not thought to prevent entry of the virus into the host cell, some anti-NA mAbs have been found to exhibit HI activity (Figure 3Aviii). Furthermore, one can imagine that—like anti-HA antibodies—anti-NA antibodies bound to the surface of infected cells may aid in their clearance by immune effector cells via antibody-dependent cell-mediated cytotoxicity (ADCC) and complement-dependent cytotoxicity (CDC) (Figure 3iv,v,vii). Conceivably, antibody independent, cell-mediated responses against NA epitopes may greatly aid in the clearance of infected cells as well. Indeed, T cells reactive to influenza protein epitopes have been described over 25 years ago [60], and recent studies in humans have attempted to more finely discern the T cell response during influenza infection and vaccination ([61] and reviewed in [62]). However, there is a paucity of information regarding the relative roles of the humoral and adaptive immune system in NA-based protection specifically.

## 4. Neuraminidase as a Vaccine Antigen

### 4.1. Immunodominance of HA over NA

It is well known that HA exhibits immunodominance over NA in influenza virus infection and vaccination [63]. In response to intramuscular (IM) vaccination with standard, trivalent subvirion (split) vaccine from the 1992–1993 season, the antibody response to NA is poor (mean seroconversion rate of 18%) when juxtaposed to that of HA (mean seroconversion rate of 84%) [13,29,63,64,65]. Yet, one may argue that this phenomenon is due to the varying and unreliable amounts of NA in vaccine formulations, which is not standardized. For instance, neither the amount nor enzymatic activity of NA was characterized in the aforementioned experiment from which the mean seroconversion rates were determined [65]. In contrast, licensed influenza vaccines are required to contain 15 µg of each HA subtype as measured by single radial immunodiffusion [38]. Couch *et al.* conducted a randomized vaccine trial on healthy young adults using six commercially available influenza virus vaccines (5 TIVs and 1 LAIV) from the 2008–2009 season to compare the immunogenicity amongst different formulations. NI titers (against VLPs containing either N1 or N2) were correlated with pre- and post-vaccination HI titers, but levels varied among different vaccine brands. The only LAIV formulation tested, Flumist, produced no NI response, while certain vaccines elicited a significantly greater response to N1 over N2 or *vice versa* [66]. The stability of the NA protein contained in vaccines could plausibly result in varied immunogenicity as well. Sultana *et al.* analyzed the stability of NA in intermediate monovalent vaccine preparations of A/California/7/2009 (H1N1), A/Victoria/210/2009 (H3N2), and B/Brisbane/60/2008 from the 2010–2011 season [67]. The enzymatic activity of the NA of each of the strains tended to be similar between vaccine lots but significantly different between strains (for instance, the enzymatic activity of the H1N1 strain was greatest when compared to that of the H3N2 and B strains). The NAs of each of the three strains behaved differently when subjected to various destabilizing agents (freeze/thaw cycles, detergents, increasing temperature). When administered IM to mice, the N1 from A/California/7/2009 (Cal09) proved to be more immunogenic than the N2 of A/Victoria/210/2009, and immunogenicity was directly proportional to enzymatic activity [67].

### 4.2. Enhancing the Immunogenicity of NA

Immunodominance can be shaped by a variety of factors, such as differences in loading of the antigen onto MHC Type II complexes or by the intrinsic preference of germline B cell populations to bind to certain antigens over others [68]. However, when HA and NA are chromatographically purified and administered to mice separately in equal molar amounts, they are equally immunogenic [69]. This suggests that the antigenic dominance may simply be due to the fact that there are more hemagglutinin molecules than neuraminidase molecules on the viral surface, which would increase loading of HA epitopes onto the MHC by sheer chance; it has been shown that HA outnumbers NA on the virion surface 4–5:1, lending support to this theory [70]. Furthermore, by measuring the extent of B and T lymphocyte proliferation *in vitro*, it has been shown that the immune response can be artificially skewed towards NA when mice are infected and then vaccinated with viruses containing homologous NA proteins but heterologous HA proteins (in this specific case, mice were infected with H3N2 and vaccinated with H7N2) [71]. To further demonstrate this “artificially skewed immunodominance” from HA to NA, the same group used a similar vaccination regimen, but in this instance measured immunologic reactivity by performing hemagglutinin inhibition (HI) and neuraminidase inhibition (NI) assays [63]; the data obtained paralleled those in the aforementioned B cell and T cell experiments. Another study showed a similar relative bias towards anti-NA seroconversion when mice were primed to N2 with infection with A/Hong Kong/1/68 (an H3N2 strain) and then boosted with purified HA or NA from A/Turkey/Mass/75-A/Aichi/2/68 (an HA-heterotypic H6N2 reassortant strain) [72]. In humans, this effect was essentially seen in a vaccination experiment involving school children in Buffalo, New York in the late 70s; the subjects were either vaccinated with an inactivated H3N2 strain, a heterotypic H7N2 reassortant strain, or placebo, and were monitored for subsequent seroconversion by HI and NI assays following a naturally occurring outbreak of the H3N2 strain, A/Port Chalmers/74. Children vaccinated with the exotic H7N2 strain showed a mean 4-fold greater increase in N2 seroconversion than those vaccinated with H3N2 and exhibited a reduced attack rate of the Port Chalmers strain. However, the protection conferred by the homotypic H3N2 was greater overall (as measured by the change in attack rate of A/Port Chalmers/74) [73].

Interestingly, the current strategy for designing a universal influenza vaccine aims to skew the humoral immune response in a similar fashion as some of the aforementioned experiments; in this case, however, the goal is to boost antibodies to the highly conserved stalk domain of HA, which is appreciably less immunogenic than the globular head domain but much more conserved across influenza virus subtypes [25,74]. This is achieved by administering successive vaccinations using influenza viruses that have been genetically engineered to express chimeric HAs, so that each subsequent immunization contains HA antigen that has an identical stalk domain—but a differing exotic globular head domain—from the previous vaccination (in this instance, the term exotic refers to HA subtypes that do not normally circulate in the human population) [19,75,76,77,78,79,80,81,82]. Presumably, if these immunizations contain identical neuraminidases, the humoral response to NA may be boosted as well, which may lead to added protection. This phenomenon has yet to be explored in the context of vaccination with chimeric viruses.

### 4.3. NI Seroconversion Rates in Modern Human Vaccine Trials

Modern human vaccine studies (summarized in Table 1) have actually shown promising NI seroconversion rates. In one such study, sera were analyzed from patients in a randomized phase I/II clinical trial using an H5N1 whole-virus inactivated vaccine produced in Vero cell culture [83]. Vaccinations were standardized based on the amount of HA, as is done with currently administered inactivated vaccines. Anti-NA titers were determined using a variety of methods, but inhibition in ELLA was found to be most sensitive. All of the 83 individuals studied had a baseline NI measurement, as was expected given the age range of the subjects (18–45 years of age) and the fact that seasonal H1N1 strains normally circulate in the population. Of greater interest is that the seroconversion rate (as defined by a four-fold or greater increase of anti-NA titers relative to baseline) after the first vaccination with H5N1 was 59.9% using ELLA, indicating that the individuals had basically all been primed with exposure to the N1 circulating in the population [83]. The same group conducted a large scale safety trial with the Vero cell-derived, inactivated H5N1 vaccine in 675 children and found seroconversion rates as high as 100% against recombinant NA from A/Vietnam/1203/2004 (see Table 1, reference [84]). A recent vaccine trial of 284 adults yielded comparably high NI seroconversion rates against the N9 neuraminidase (an average seroconversion rate as high as 97.2% was seen in subjects who were vaccinated with 5 mg HA-standardized VLPs supplemented with ISCOMATRIX adjuvant) (see Table 1, reference [85]). Perhaps such high seroconversion rates can be attributed to the stability of the NA protein in whole virus inactivated or VLP-based preparations.

**Table 1:** NA-based vaccination studies.

Technology	Model	Main Findings	Ref.
X-32 (H7N2) reassortant strain vaccine	Humans*	Texan prisoners immunized with an H7N2 reassortant strain displayed significantly lower nasal wash titers when challenged with an infectious dose of circulating H3N2 than those immunized with influenza B virus and the nasal wash titers were inversely proportional to serum anti-NA titer	[89]
X-42 (H7N2) reassortant strain vaccine	**Humans**	School children from Buffalo, NY immunized with an H7N2 reassortant strain displayed a lower attack rate of naturally circulating H3N2 virus than those receiving placebo vaccination	[73]
N2 purified from A/Beijing/32/92 virus	Mice, rabbits, and **humans**	NA was isolated from virus disrupted with detergent and purified (by affinity chromatography using oxamic acid agarose) treated with formalin, and tested for enzymatic activity and immunogenicity in BALB/c mice, rabbits, and humans. The vaccine was shown to be immunogenic and non-toxic, overall	[98,99]
N2 from A/Victoria/3/75 expressed in yeast	Mice	Mice were protected against a lethal challenge of influenza virus after immunization with a recombinant head domain of NA, expressed in the yeast species *P. pastoris*	[100]
N2 DNA vaccine	Mice	Mice immunized with NA-expressing plasmid DNA were protected against lethal challenge with homologous and heterologous H3N2 strains, but failed to be protected from H1N1 challenge	[101,102]
TIV supplemented with chromatographically purified N1 and N2	Mice	Mice immunized with TIV supplemented with chromatographically purified N1 and N2 showed a greater reduction in viral lung titers than those receiving TIV alone after challenged with heterotypic strains	[95]
Soluble NA produced in a cell-free system	Ferrets	Immunization with soluble tetrameric NA reduces signs of morbidity after influenza virus infection (such as bodyweight loss and lung pathology) upon challenge with a homologous H1N1 strain	[103]
A/Vietnam/1203/2004 (H5N1) whole-virus inactivated vaccine produced in Vero cell culture	**Humans**	Phase I/II clinical trials of individuals vaccinated with H5N1 produced in Vero cell culture displayed an average NI seroconversion rate of 59.9% against N1	[83]
Recombinant, hypoglycosylated NA produced in the yeast species, *Pichia pastoris*	Mice	Mice immunized with a recombinant, hypoglycosylated form of NA showed higher NI titers than those immunized with an equivalent form of hyperglycosylated NA	[97]
H5N1 VLPs (produced by baculovirus infected insect cells)	Mice	Mice immunized with H5N1 VLPs derived from the avian influenza virus strain RG14 H5N1 showed robust NI seroconversion and *in-vivo* protection against lethal challenge with the matched H5N1 strain and Cal09 H1N1, but were not protected from challenge with PR8 H1N1	[104]
293T-produced VLPs containing NA and matrix proteins from H1N1	Mice	Immunization of VLPs protects mice from lethal infection with H5N1	[105]
VLPs containing the N1 and M1 from PR8 (produced by baculovirus-infected insect cells)	Mice	Mice immunized intranasally with VLPs containing N1 and M1 were completely protected against a lethal challenge with the matched, homologous PR8 strain (H1N1) and against a heterosubtypic H3N2 strain (A/Philippines/2/82), although the mice challenged with the H3N2 virus were not protected from weight loss	[106]
H7N9 VLPs (produced by baculovirus-infected insect cells)	**Humans**	Humans immunized with H7N9 (A/Anhui/1/13) VLPs supplemented with ISCOMATRIX adjuvant displayed an average NI seroconversion rate of 97.2% against N9	[85]
Intramuscular immunization with seasonal split TIV	Ferrets	Ferrets that received two intramuscular vaccinations with split TIV vaccines were protected against lethal challenge with H5N1	[107]
Mouse monoclonal antibodies	Mice	Mice prophylactically given one dose of broadly reactive N1 mAbs were protected against lethal challenge with seasonal H1N1, pandemic 2009 H1N1, and H5N1	[48]
Two priming immunizations with A/Vietnam/1203/2004 (H5N1) whole-virus inactivated vaccine produced in Vero cell culture followed by a boost of A/Indonesia/05/2005	**Humans**	Test of safety and efficacy of a previously mentioned Vero cell-derived H5N1 vaccine [83] in 675 children aged 6 months–17 years showed no adverse effects and 93.1%–100% of subjects displayed MN titers of 1:20 or greater against the H5N1. NI titers were induced in greater than 90% of subjects.	[84]

* Clinical data in humans is listed in bold.

### 4.4. Historic Evidence of NA-Based Protection in Humans

**Figure 5:**
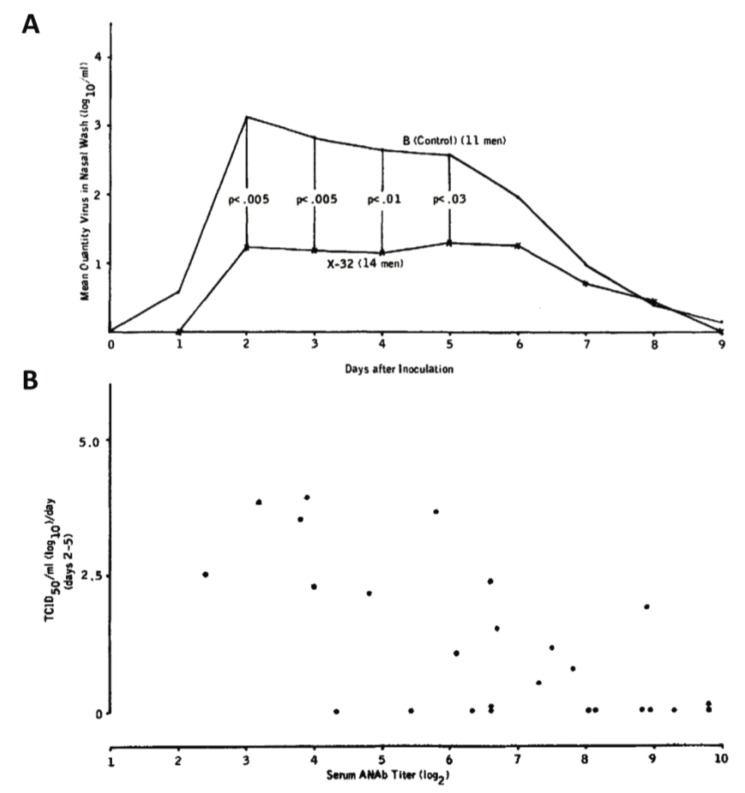
Historic evidence of NA-based protection in human vaccine experiments. (**A**) Male prisoners vaccinated with X-32 (H7N2) displayed significantly less nasal wash titers when intranasally challenged with H3N2 than those who were vaccinated with influenza B virus. (**B**) Level of virus in nasal wash specimens was inversely proportional to serum anti-NA antibody titer (taken with permission from Couch *et al.*, 1974) [89].

While antibodies against NA may not be neutralizing, they have been shown to reduce viral lung titers to sub-pathogenic levels in mouse models [86]. Humans challenged with antigenically drifted HA but homologous NA exhibit fewer days of viral shedding, less severe symptoms, and reduced viral shedding overall [87]. In fact, some would argue that this so called “permissive immunity” is advantageous to the host, as viral replication allows for the activation of the innate immune system, and could lead to increased antigenic presentation to T cells. The protection afforded by NA as an antigen has been intimated in early epidemiological studies in humans, including the aforementioned study of Buffalo school children [73] as well as a study of adults between the ages of 20–45 in Tecumseh, MI, which showed a correlation between the prior existence of cross-reactive NA antibodies and reduced disease severity from naturally circulating Hong Kong/68; this effect seemed to be independent of the presence of HA antibodies [88]. In yet another early human vaccine experiment which would probably not pass current IRB guidelines, 39 male prisoner volunteers from the Texas Department of Corrections were first immunized with either X-32 (an H7N2 reassortant virus, in which the neuraminidase is derived from A/Aichi/2/68) or influenza B virus (as a control) and then intranasally challenged with a purified live isolate of circulating 1970 H3N2 virus [89]. The group that received the X-32 vaccination featuring a homologous NA developed statistically significantly less cases of infection. Moreover, while almost 100% of those with confirmed influenza infection in the control group displayed febrile illness, this was only the case for less than half of those with infection in the X-32 group. In general, men in the control group exhibited a greater magnitude of viral shedding (although there was no appreciable difference in length of viral shedding) and—overall—anti-NA titers tended to correlate with decreased viral shedding and decreased severity of infection (Figure 5) [89]. Please refer to Table 1 for a summary of these findings.

### 4.5. Anti-NA Antibodies Affect Viral Evolution

Understandably, antibodies against the neuraminidase may influence the dynamics and direction of influenza virus evolution. In fact, it has been hypothesized that the sudden herd immunity elicited by cross reactive N2 antibodies in the human population was the driving force behind the disappearance of circulating pandemic Asian Influenza virus strains (H2N2) once the pandemic Hong Kong influenza virus (H3N2) was introduced into the population in 1968 [90]. Furthermore, while NA drift rates are expectedly slower than that of HA due to less immune pressure (0.45%–1% *vs.* 1%–2% respectively) [44,91], there is evidence that NA drift may be directional due to the frequency of amino acid change in immunologically relevant epitopes. For instance, after a mouse Fab-N2 crystal structure was solved, six out of 11 of the amino acid side chain contacts on the epitope binding surface were shown to have changed over 10 years. This is significant considering one report, which found that 90% of the nucleotide positions and 87% of the amino acid residues in N2 remained invariant over the course of 22 years [92]. The comparatively higher rate of the aforementioned Fab-mapped epitope would suggest that it is in fact relevant to human immunity and that NA drift (like that of HA) undergoes selective pressure from the human immune system [93]. Indeed, this interpretation has gained increasing support in the new age of high throughput sequencing and large-scale phylogenetic analysis. Such analysis on the evolutionary history of influenza A NA has revealed that positively selected amino acid sequence changes expectedly correspond with residues on the 3D structure that are known to be involved in B cell antigenicity [94].

### 4.6. Future Considerations in the Design of NA-Based Vaccines

It is well recognized in the NA field that a vaccine that purely raises antibodies to neuraminidase is not desirable and would not be as effective as one which includes some combination of the NA and HA antigen [13]. Thus, studies have aimed to show the effectiveness of vaccines supplemented with the NA antigen. Indeed, TIV supplemented with chromatographically purified N1 and N2 was shown to be more effective than TIV alone in reducing pulmonary viral titers in mice following infection with heterotypic HA strains [95]. Proponents of NA vaccines argue that the amount of NA in existing TIV formulations should at least be standardized, a feat which could be accomplished by straightforward and available enzyme assays [38]. In an era of new recombinant technologies, scientists have become more creative in their development of potential NA vaccines and broadly-inhibiting, anti-NA mAbs (Table 1 and Table 2). Novel approaches have raised new questions or challenged older dogma concerning current influenza vaccine design; one question raised by the study of recombinant baculovirus-produced NA is if intact enzymatic activity is necessary for NA immunogenicity [13,28,38]. If so, it would be important for recombinantly-produced NA to be tetrameric and enzymatic function would have to be confirmed prior to use. Interestingly, elderly individuals who received a higher dosage of the standard seasonal TIV (containing 60 µg of HA instead of the traditional 15 µg and eight times higher neuraminidase activity) displayed significantly higher mean anti-N1 and anti-N2 titers post-vaccination than those receiving the traditional seasonal TIV [96]. The glycosylation pattern of the NA may also foreseeably alter its immunogenicity. A hypoglycosylated form of recombinant NA produced in a mutant yeast species of *Pichia pastoris* with a defective α-1,6-mannosyltransferase gene was shown to induce significantly higher NA titers in mice (as measured by ELISA) than the equivalent amount of hyperglycosylated NA produced in the wild type yeast species [97].

**Table 2:** Isolated anti-NA monoclonal antibodies.

Name	Binding Profile	Organism/Method of Isolation	IC50 Range	Key Findings	Ref.
2B9	N1 NAs from highly pathogenic H5N1 avian influenza viruses as well as N1 NAs from H1N1 strains	Mouse mAb—mice were immunized with recombinant N1 NA from A/Vietnam/1194/2004 and antibody was isolated using hybridoma technology	<1–250 µg/mL (as measured by NI)	2B9 was shown to enzymatically inhibit NAs from both homologous and heterologous H5N1 strains, as well as some H1N1 strains. Mice passively immunized with 2B9 were 50% protected against lethal challenge with a closely related H5N1 virus	[115]
3A2, 4G2, 1H5, 2D9, *and several others*	N1 NA from A/Brisbane/59/2007 (seasonal H1N1), Cal09 (pandemic H1N1) and A/Vietnam/1203/2004 (avian H5N1)	Mouse mAb—mice were immunized twice intranasally with A/Brisbane/59/2007 and boosted a third time with purified virus	7.7 ng/mL–>32,000 ng/mL (as measured by NI)	Some of the mAbs isolated were broadly reactive against the N1 of seasonal H1N1, pandemic 1918 H1N1, pandemic 2009 H1N1, and H5N1 viruses. A single dose of one mAb, 3A2, was able to fully protect mice against lethal challenge with seasonal and 2009 pandemic H1N1 viruses and resulted in significant protection against wild-type H5N1	[48]
HCA-2 MAb	Universally conserved 9-amino acid long NA sequence, ILRTQESEC	Rabbit mAb—9 amino acid long peptide (ILRTQESEC) was conjugated to a 6-aminocaproic-lysine-lysine-cysteine linker and used to immunize rabbits. Antibodies were produced using hybridoma technology by a commercial contract research organization.	2.56–18.2 µg/mL (as measured by growth inhibition of various viral strains) 0.95–181.93 µg/mL (as measured by NI of representative NA subtypes)	HCA-2 mAb was shown to inhibit the enzymatic activity of practically all known influenza A NA subtypes (N1-9) as well as those from both the Yamagata and Victoria influenza B virus lineages	[46,47]

### 4.7. Evidence of NA-Based Heterologous Protection

The jury is still out as to whether or not NA can elicit heterosubtypic immunity (across NA subtypes). Even the extent to which NA can elicit heterologous protection within a subtype is poorly characterized, although the consensus is that immunologic exposure to NA can at least confer cross-protection to lethal challenge with heterologous strains if the NA from the challenge strain and the NA received in vaccination share high sequence homology. In one such study, chickens vaccinated with a virus-vectored vaccine expressing N2 resembling the challenge virus vaccine (98% amino acid identity) were 88% protected against mortality whereas those immunized with a more distant N2 (85% amino acid identity) were only 25% protected [108]. Similarly, mice vaccinated with N2 DNA failed to be protected against heterosubtypic H1N1 challenge, but were protected against heterologous H3N2 challenge [102]. In a more recent VLP study, mice received two successive vaccinations of either H5N1-VLP, recombinant H5N1-VLP (in which the M2 ectodomain was fused to the N terminus of HA), or N1-VLP (a VLP which was shown only to contain NA on the surface). The NA used in each of these VLPs was from RG14, an avian-derived strain (H5N1). In this experiment, robust NI seroconversion and *in-vivo* protection was seen in mice lethally challenged with the matched H5N1 strain and with Cal09 (H1N1), but not in those lethally challenged with A/Puerto Rico/8/34 (H1N1) [104]. Expectedly, the NA of RG14 is phylogenetically more closely related to that of (Cal09) than to that of A/Puerto Rico/8/34 (PR8) (Figure 1B). Marcelin *et al.* attempted to characterize the degree of cross-reactivity and possible cross-protection of antibodies against the NA from seasonal H1N1 strains to that of the 2009 H1N1 pandemic strain. Indeed, this group had previously shown that the sera of elderly patients (aged 70 and older) who received either the 2007–2008 or 2008–2009 seasonal TIV displayed significant cross-reactivity (as measured by NI) to the pandemic H1N1 strain A/Tennessee/1-560/2009 [109]. The NAs from the seasonal strains included in the 2007–2008 and 2008–2009 TIV formulations (A/Solomon Islands/3/06 and A/Brisbane/59/2007, respectively) share 84% amino acid sequence identity to the NA of Cal09, which is 99% similar to A/Tennessee/1-560/2009. Using reverse genetics, Marcelin *et al.* engineered each of these respective viruses with an internal PR8 backbone to study cross-reactivity and cross-protection in mice, achieving mixed results. Mice immunized with either the PR8-reassortant Brisbane or Solomon Island seasonal H1N1 strains displayed cross-reactivity to Cal09 NA (as measured by ELISA binding to the baculovirus-produced Cal09 NA ectodomain), although these titers were 16–32 fold less than those of the matched positive controls (immunization with Cal09). Modest cross-protection was seen when these mice were challenged with a lethal dose of Cal09; a higher percentage of mice were protected compared to the PBS control group, but this equated to only ~40%–50% of all mice. Little change was seen in weight loss kinetics and no significant reduction was seen in viral lung titers [110]. In another demonstration of cross-reactivity, Xie *et al.* analyzed human sera from individuals who received the A/New Jersey/76 (the NA of which displays 82% amino acid identity to that of Cal09) monovalent inactivated vaccine in 1976 clinical trials and found significant induction of NA titers post vaccination (as measured by NI) against both the A/New Jersey/76 NA and the Cal09 NA (which are relatively distant, as shown in Figure 1B). When passively transferred to mice, these human sera protected against lethal challenge with a reassortant virus containing the Cal09 NA but a mismatched HA, suggesting NA-based heterologous protection [111]. Please refer to Table 1 for a summary of these findings.

It is important to realize that, while genetic changes and antigenic changes of NA may be tightly correlated, genetic change alone is not necessarily a reliable predictor of antigenic change. The process of antigenic cartography is able to enhance the understanding of viral evolution by mapping antigenic distance using available data on the antigenic properties of the virus. Extensive antigenic maps of HA created by utilizing data on hemagglutinin inhibition (HI) have revealed that the antigenic evolution of the viral glycoprotein is not as gradual as phylogenetic changes in its nucleic or amino acid sequences. Small changes in sequence (such as one amino acid substitution) may correspond to large changes in antigenicity, and *vice versa* [112]. Of interest, antigenic maps of N1 and N2 from the influenza virus strains used in 15 years of seasonal vaccine formulations were created using NI endpoint titers and strikingly revealed discordant antigenic evolution between HA and NA [113]. What is more, while the rate of nucleic acid change in the sequence of NA was continuous, the rate of antigenic change was more abrupt, with periods of stasis lasting over a decade (1991–2006), followed by a sudden change in the A/Brisbane/59/2007 strain. Using mutagenesis, the group that performed the aforementioned study was able to attribute this change in antigenicity to one amino acid substitution [113]. However and in general, one must be reminded that antigenic cartography data is generated using sera from ferrets that are infected with the strain of interest but which lack pre-existing immunity against influenza viruses which might highly impact the immune response generated; understandably, this response may differ in humans.

### 4.8. Evidence of NA-Based Heterosubtypic Protection

There are far fewer examples of NA-based heterosubtypic protection in the vaccine literature, if any. In one recent study, mice immunized intranasally with VLPs (produced via baculovirus-infected Sf9 cells) containing the NA and M1 protein from PR8 (H1N1) were completely protected against a lethal challenge with the matched, homologous PR8 strain (H1N1) and against a heterotypic H3N2 strain (A/Philippines/2/82), although the mice challenged with the H3N2 virus were not protected from weight loss. Furthermore, mice infected with PR8 displayed a near 4 log decrease in viral lung titers, while those challenged with A/Philippines/2/82 displayed a near 2 log decrease [106]. While any evidence of NA-based heterosubtypic protection is exciting, it is difficult to conclude that the breadth of this protection was due to NA alone, as the M1 protein from PR8 was present in the VLPs as well.

### 4.9. Antibodies against H1N1 Protect against H5N1 in Multiple Animal Models

Pivotal vaccine experiments in animals in the past few years have demonstrated the exciting potential of NA antibodies against H1N1 to protect against lethal H5N1 challenge and *vice versa*. Such cross-protection could be utilized in the event of an emergent H5N1 pandemic strain. In one experiment, pigs previously infected with an H1N1 virus exhibited cross-reactive NI titers to H5N1 and were subsequently protected when challenged with H5N1 virus; additionally, none of the pigs in the H1N1 infected group displayed clinical symptoms of flu infection such as cough, fever, and tachypnea [114]. Using hybridoma technology, one group immunized mice with recombinant NA from A/Vietnam/1194/2004 (H5N1) and isolated a mAb that was able to enzymatically inhibit NAs from both homologous and heterologous H5N1 strains, as well as those from H1N1 strains (see Table 2). Mice that received this mAb intravenously demonstrated 50% protection from a lethal challenge with a closely related H5N1 virus [115]. More recently in mice, intranasal immunization with VLPs containing NA and matrix proteins from 2009 pandemic H1N1 was able to protect against lethal H5N1 challenge [105]. In ferrets, two IM vaccinations of seasonal TIV was protective against lethal challenge with H5N1, and this protection was correlated with the presence of NA antibodies (based on NI assays) [107]. These findings were molecularly fortified, when one group used mouse monoclonal antibodies against N1 to map the conserved NA epitopes shared between seasonal H1N1, pandemic H1N1, and avian H5N1 [48].

The mAbs were initially raised against A/Brisbane/59/2007 (H1N1), but epitope mapping revealed that the amino acid residues 273, 338, and 339 in N1 (which were part of 12 essential residues for binding of all of the mAbs) were located in the enzymatic active site and conserved in the N1 of seasonal and avian strains. As a proof of concept, some of the mAbs were broadly reactive against the N1 of seasonal H1N1, pandemic 1918 H1N1, pandemic 2009 H1N1, and H5N1 viruses. Moreover, a single dose of these broadly reactive mAbs (administered prophylactically) was able to fully protect mice against lethal challenge with seasonal and 2009 pandemic H1N1 viruses and resulted in significant protection against wild-type H5N1 (see Table 2, reference [48]). These recent developments are crucial given the constant threat of emerging pandemic strains.

## 5. Conclusions

In some ways, viruses like influenza (with only eight RNA segments and 11 encoded proteins) seem exceedingly simple, yet the complex interplay of viruses and host immunity is perhaps nature’s finest example of co-evolution, and continues to shed light on principles of immunity, ecological selection, and cellular signaling pathways. As viruses mutate to escape the immune response, a natural genetic phenomenon seems to personify a desperate quest to achieve perpetual existence. In the age of modern medicine, humans have devised countless ways to wage war on infectious disease in attempts to progress human health.

In the case of the influenza A virus, the majority of efforts are aimed in producing vaccines that elicit broader spectra of HA antibody coverage. However, as demonstrated, there is exceeding potential in using the NA as a vaccine antigen. Further, while several studies have aimed to elucidate the spectrum of NA-based protection, a slew of questions remain unanswered. Most of the animal studies on NA vaccines are restricted to mice and little work has been done using ferrets, the prime animal model for influenza virus transmission. Could a vaccine that raises a robust response against NA affect viral transmission in the ferret model? As anti-NA antibodies may prevent detachment of viral particles from host cell, such a hypothesis seems plausible. Furthermore, augmenting the humoral response to NA will expectedly increase the selective pressure of viruses in nature. How will the escape of NA from such a response affect its enzymatic activity or its inhibition by current antiviral drugs, and will such drift lead to more drug resistant viruses? What are the implications in NA’s enzymatic activation of TGF-b, if any, and should these be considered more closely when brainstorming methods of NA vaccine design? These questions and others remain as researchers continue the quest towards a more effective influenza virus vaccine.

## Supplementary Files

Supplementary File 1 Supplementary Information (PDF, 285 KB)
