# Supplementary material for: In the Shadow of Hemagglutinin: A Growing Interest in Influenza Viral Neuraminidase and Its Role as a Vaccine Antigen
Source: Viruses. 2014 Jun 23;6(6):2465–94. doi: 10.3390/v6062465 (PMC4074938; doi:10.3390/v6062465)
Supplement: Supplementary File 1 — Supplementary Information (PDF, 285 KB) [file viruses-06-02465-s001.pdf]

Supplementary Information

Table S1.

Strain Name	Abbreviation	Subtype	Accession Number	Database
**A/Anhui/1/2013**	N9	H7N9	EPI439509	GISAID
A/Beijing/353/1989	BJ89	H3N2	CY121026.1	Genbank
A/Bilthoven/552/1973	BH73	H3N2	CY113095.1	Genbank
A/Brevig Mission/1/1918	BM18	H1N1	AF250356.2	Genbank
A/Brisbane/59/2007	Bris07	H1N1	CY163866.1	Genbank
**A/California/04/2009**	N1, Cal09	H1N1	FJ969517.1	Genbank
A/canine/Jiangsu/06/2010	canineJS10	H3N2	JN247621.1	Genbank
A/chicken/El Salvador/102711-2/2001	chickES01	H5N2	GU052638.1	Genbank
A/chicken/Hong Kong/WF126/2003	chickHK03	H9N2	AY664709.1	Genbank
A/chicken/Hunan/1/2012	chickHunan12	H9N2	KF714777.1	Genbank
A/chicken/Italy/4789/1999	chickIT99	H7N1	CY025127.1	Genbank
A/chicken/Korea/LPM88/2006	chickKorea06	H3N2	EU301301.1	Genbank
A/chicken/New Jersey/13839-17/1995	chickNJ95	H2N2	CY117053.1	Genbank
A/common teal/Netherlands/10/2000	ctNL00	H1N1	CY060180.1	Genbank
A/Cottbus/1/1964	Cb64	H2N2	CY032263.1	Genbank
A/Denver/1957	DV57	H1N1	CY008990.1	Genbank
**A/duck/Eastern China/01/2007**	N6	H4N6	EU429790.1	Genbank
A/duck/Potsdam/1402-6/1986	duckPD86	H5N2	GU052542.1	Genbank
A/England/42/1972	Eng76	H1N1	CY113087.1	Genbank
A/flat-faced bat/Peru/033/2010	N11	H18N11	CY125947.1	Genbank
A/Fort Monmouth/1/1947	FM47	H1N1	CY009614.1	Genbank
**A/Hong Kong/1/1968**	N2, HK68	H3N2	CY112251.1	Genbank
A/Indiana/10/2011	Ind11	H3N2	JQ070791.1	Genbank
**A/Jiangxi-Donghu/346/2013**	N8	H10N8	EPI497479	GISAID
A/little yellow-shouldered bat/Guatemala/060/2010	N10	H17N10	CY103894.1	Genbank
**A/mallard/California/1305/2010**	N7	H10N7	CY094767.1	Genbank
A/mallard/Sweden/24/2002	N4	H8N4	CY064796.1	Genbank
**A/mallard/Sweden/86/2003**	N5	H12N5	CY060392.1	Genbank
A/New Caledonia/20/1999	NC99	H1N1	CY033624.1	Genbank
A/New Jersey/8/1976	NJ76	H1N1	CY130120.1	Genbank
A/Panama/2007/1999	Pan99	H3N2	CY112919.1	Genbank
A/Perth/16/2009	Perth09	H3N2	CY081429.1	Genbank
A/Philippines/2/1982	Phil82	H3N2	CY121247.1	Genbank
A/Poland/5/1967	Po67	H3N2	AY209926.1	Genbank
A/Puerto Rico/8/34/Mount Sinai	PR8	H1N1	AF389120.1	Genbank
A/rhea/North Carolina/39482/1993	rheaNC99	H7N1	EF607926.1	Genbank
A/Singapore/1/1957	SG57	H2N2	AB124654.1	Genbank
A/swine/Iowa/15/1930	swineIA30	H1N1	EU139833.1	Genbank
A/swine/Jiangsu/40/2011	swine JS11	H1N1	JQ319650.1	Genbank
**A/swine/Missouri/4296424/2006**	N3	H2N3	EU258945.1	Genbank
A/swine/North Carolina/SG1175/2003	swineNC03	H1N1	CY159551.1	Genbank

Table S1. *Cont.*

Strain Name	Abbreviation	Subtype	Accession Number	Database
A/Taiwan/1/1986	TW86	H1N1	KF356051.1	Genbank
A/Texas/36/1991	Tx91	H1N1	CY033600.1	Genbank
A/Texas/50/2012	Tx12	H3N2	KC892281.1	Genbank
A/USSR/90/1977	USSR77	H1N1	CY121880.1	Genbank
A/Viet Nam/1203/2004	VN04	H5N1	HM006761.1	Genbank
A/WS/1933	WS33	H1N1	L25816.1	Genbank
B/Beijing/184/**19**93	Beijing93	B	AJ784090.1	Genbank
B/Brisbane/60/2008	Bris08	B	FJ766841.1	Genbank
B/Florida/4/2006	Flor06	B	CY033878.1	Genbank
B/Hong Kong/05/1972	HK72	B	DQ508923.1	Genbank
B/Hong Kong/06/2001	HK01	B	CY018631.1	Genbank
B/Lee/1940	Lee40	B	CY115113.1	Genbank
B/Malaysia/2506/2004	Mal04	B	CY038289.1	Genbank
B/Maryland/1959	MD59	B	M30633.1	Genbank
B/Massachusetts/04/2013	Mass13	B	KJ136226.1	Genbank
B/Memphis/19/1996	Memphis96	B	AF129920.2	Genbank
B/Nevada/01/2014	Nevada14	B	KJ532168.1	Genbank
B/Panama/45/1990	Pan90	B	CY018351.1	Genbank
B/Russia/1969	Rus69	B	EF626638.1	Genbank
B/Shanghai/361/2002	SH02	B	EF541477.1	Genbank
B/Sichuan/379/1999	Sichuan99	B	AY139080.1	Genbank
B/Switzerland/4291/1997	Swiss97	B	AY191501.1	Genbank
B/Victoria/2/1987	Vic87	B	AB036870.1	Genbank
B/Vienna/1/1999	Vienna99	B	AY191500.1	Genbank
B/Wisconsin/01/2010	Wisc10	B	CY115185.1	Genbank
**B/Yamagata/16/1988**	NB, Yam88	B	AY139081.1	Genbank

© 2014 by the authors; licensee MDPI, Basel, Switzerland. This article is an open access article distributed under the terms and conditions of the Creative Commons Attribution license (http://creativecommons.org/licenses/by/3.0/).
